# Associations between unit workloads and outcomes of first extubation attempts in extremely premature infants below a gestational age of 26 weeks

**DOI:** 10.3389/fped.2023.1090701

**Published:** 2023-03-17

**Authors:** Mari Oma Ohnstad, Hans Jørgen Stensvold, Are Hugo Pripp, Christine Raaen Tvedt, Lars-Petter Jelsness-Jørgensen, Henriette Astrup, Beate Horsberg Eriksen, Mai Linn Lunnay, Khalaf Mreihil, Tanja Pedersen, Siren Irene Rettedal, Terje Reidar Selberg, Rønnaug Solberg, Ragnhild Støen, Arild Erland Rønnestad

**Affiliations:** ^1^ Department of Master and Postgraduate Education, Lovisenberg Diaconal University College, Oslo, Norway; ^2^ Faculty of Medicine, University of Oslo, Oslo, Norway; ^3^ Department of Neonatal Intensive Care, Division of Pediatric and Adolescent Medicine, Oslo University Hospital, Oslo, Norway; ^4^ Oslo Centre of Biostatistics and Epidemiology, Research Support Services, Oslo, Norway; ^5^ Faculty of Health Sciences, OsloMet – Oslo Metropolitan University, Oslo, Norway; ^6^ Department of Health and Welfare, Østfold University College, Halden, Norway; ^7^ Department of Internal Medicine, Østfold Hospital Trust, Kalnes, Norway; ^8^ Department of Pediatric and Adolescent Medicine, Sorlandet Hospital Trust, Kristiansand, Norway; ^9^ Department of Pediatrics, Møre and Romsdal Hospital Trust, Ålesund, Norway; ^10^ Clinical Research Unit, Norwegian University of Science and Technology, Trondheim, Norway; ^11^ Department of Pediatrics and Adolescence Medicine, University Hospital of North Norway, Tromsø, Norway; ^12^ Department of Pediatrics and Adolescence Medicine, Akershus University Hospital, Lørenskog, Norway; ^13^ Neonatal Intensive Care Unit, Department of Pediatrics, Haukeland University Hospital, Bergen, Norway; ^14^ Department of Pediatrics, Stavanger University Hospital, Stavanger, Norway; ^15^ Faculty of Health Sciences, University of Stavanger, Stavanger, Norway; ^16^ Department of Pediatrics and Adolescence Medicine, Østfold Hospital Trust, Kalnes, Norway; ^17^ Department of Pediatrics, Vestfold Hospital Trust, Tønsberg, Norway; ^18^ Department of Pediatric Research, Oslo University Hospital, Oslo, Norway; ^19^ Department of Neonatology, St Olavs – Trondheim University Hospital, Trondheim, Norway; ^20^ Department of Clinical and Molecular Medicine, Norwegian University of Science and Technology, Trondheim, Norway; ^21^ Research Group for Clinical Neonatal Medicine and Epidemiology, Department of Neonatal Intensive Care, Division of Pediatric and Adolescent Medicine, Oslo University Hospital, Oslo, Norway

**Keywords:** extremely premature infants (EP infants), extubation, workload, weekend effect, resillience, mechanical ventilatioin

## Abstract

**Objective:**

The objective was to explore whether high workloads in neonatal intensive care units were associated with short-term respiratory outcomes of extremely premature (EP) infants born <26 weeks of gestational age.

**Methods:**

This was a population-based study using data from the Norwegian Neonatal Network supplemented by data extracted from the medical records of EP infants <26 weeks GA born from 2013 to 2018. To describe the unit workloads, measurements of daily patient volume and unit acuity at each NICU were used. The effect of weekend and summer holiday was also explored.

**Results:**

We analyzed 316 first planned extubation attempts. There were no associations between unit workloads and the duration of mechanical ventilation until each infant’s first extubation or the outcomes of these attempts. Additionally, there were no weekend or summer holiday effects on the outcomes explored. Workloads did not affect the causes of reintubation for infants who failed their first extubation attempt.

**Conclusion:**

Our finding that there was no association between the organizational factors explored and short-term respiratory outcomes can be interpreted as indicating resilience in Norwegian neonatal intensive care units.

## 1. Introduction

Neonatal intensive care units (NICUs) provide care for some of the most vulnerable patients admitted to hospitals, and all admissions to Norwegian NICUs are essentially emergencies (1).

In Norway, the vast majority of extremely premature (EP) infants who are born alive between 23 and 26 weeks of gestational age (GA) receive transitional assistance, mainly with respiratory support. Those who respond positively are admitted to a NICU immediately after birth. Survival rates decrease with decreasing GA, and the smallest babies require treatment in the NICU for weeks or months (2).

The risk of neonatal mortality and morbidity has been shown to increase with increased workloads and decreased staff ratios (3–5). Moreover, NICUs differ from adult and pediatric intensive care units, which exclusively treat intensive care patients, as NICUs treat patients with varied resource needs, from highly intensive care to nearly normal maternity care (6). This creates challenges regarding unit staffing (7). Synnes et al. linked intraventricular hemorrhage to unit characteristics, suggesting that practices in NICUs with higher patient volumes and those with higher neonatologist-to-house staff ratios result in a lower incidence of severe intraventricular hemorrhage (8). In a study from Canada, greater resource use in the unit at the time of admission was associated with a higher risk of neonatal morbidity in very premature infants (9). Furthermore, weekends and holidays have been identified as times when staffing in hospitals tends to be lower, and several researchers have noted associations between weekend admissions and worse patient outcomes (10–12).

For most EP infants <26 weeks GA, mechanical ventilation (MV) is required for survival (13, 14). However, MV itself is associated with complications (15, 16), and the optimal timing of extubation is one of many challenges for clinicians (17). Close and continuous observation and monitoring, as well as clinical assessment, are essential to decide whether and why MV is needed and to prevent prolonged MV treatment or failure of an extubation attempt (18). Moreover, the post-extubation period is considered a time during which the EP infant <26 weeks GA requires special attention and management to prevent reintubation.

It is unknown whether a high unit workload affects the duration of MV until the first extubation attempt and the first extubation outcome. The main objective of this study was to explore whether high workloads in NICUs influenced timing of first planned extubation attempts or affected extubation success. We examined the association of unit workloads and the effects of weekends or summer holiday with the duration of MV until the first extubation attempt and the outcome of the first extubation attempt. As many EP infants <26 weeks GA are reintubated after their first extubation attempt (19), we explored whether high workloads in the NICUs affected respiratory morbidity for the infants reintubated within 72 h after the extubation attempt. Therefore, our secondary objective was to assess the association of unit workloads and the effects of weekends or summer holiday with indicators of respiratory morbidity before and shortly after reintubation.

## 2. Methods

### 2.1. Setting

This population-based cross-sectional study included infants born at 22^0^ through 25^6^ weeks GA and admitted to a Norwegian NICU between January 1, 2013, and December 31, 2018. The population being explored represent a subgroup of EP infants, as we included infants born before 26 weeks GA. Eligible infants were identified in the Norwegian Neonatal Network (NNN) database. An informational letter describing the purpose of the study was distributed to the infants’ mothers and included an opt-out alternative. Infants were enrolled in the study if the mother did not indicate a desire to opt out within four weeks.

### 2.2. Data collection

We examined data from the NNN supplemented by data extracted from medical records. Data on all patients admitted to any Norwegian NICU (*n* = 20) are collected daily by trained staff and entered into the NNN’s electronic registration platform. The NNN contains anthropometric and demographic data and detailed data on resuscitation, treatment modalities, treatment procedures, diagnoses, outcome parameters, and status at discharge.

From the NNN, we extracted perinatal variables, which included antenatal steroids, delivery method and plurality, and demographic variables, such as GA, sex, birth weight, and weight at GA. In addition, the Clinical Risk Index for Babies (CRIB II) and Apgar scores at 5 min were included as variables describing illness severity and general condition at birth. Furthermore, we extracted delivery room variables, which included endotracheal intubation and surfactant administration. From the medical records, we extracted data on MV settings and blood gas samples.

### 2.3. Exposure: unit workload

Unit workloads were calculated based on variables extracted from the NNN database for each day during the six years studied. To describe the workloads, measurements of daily patient volume and unit acuity at each participating NICU were derived and used as follows.

**Figure 1:**
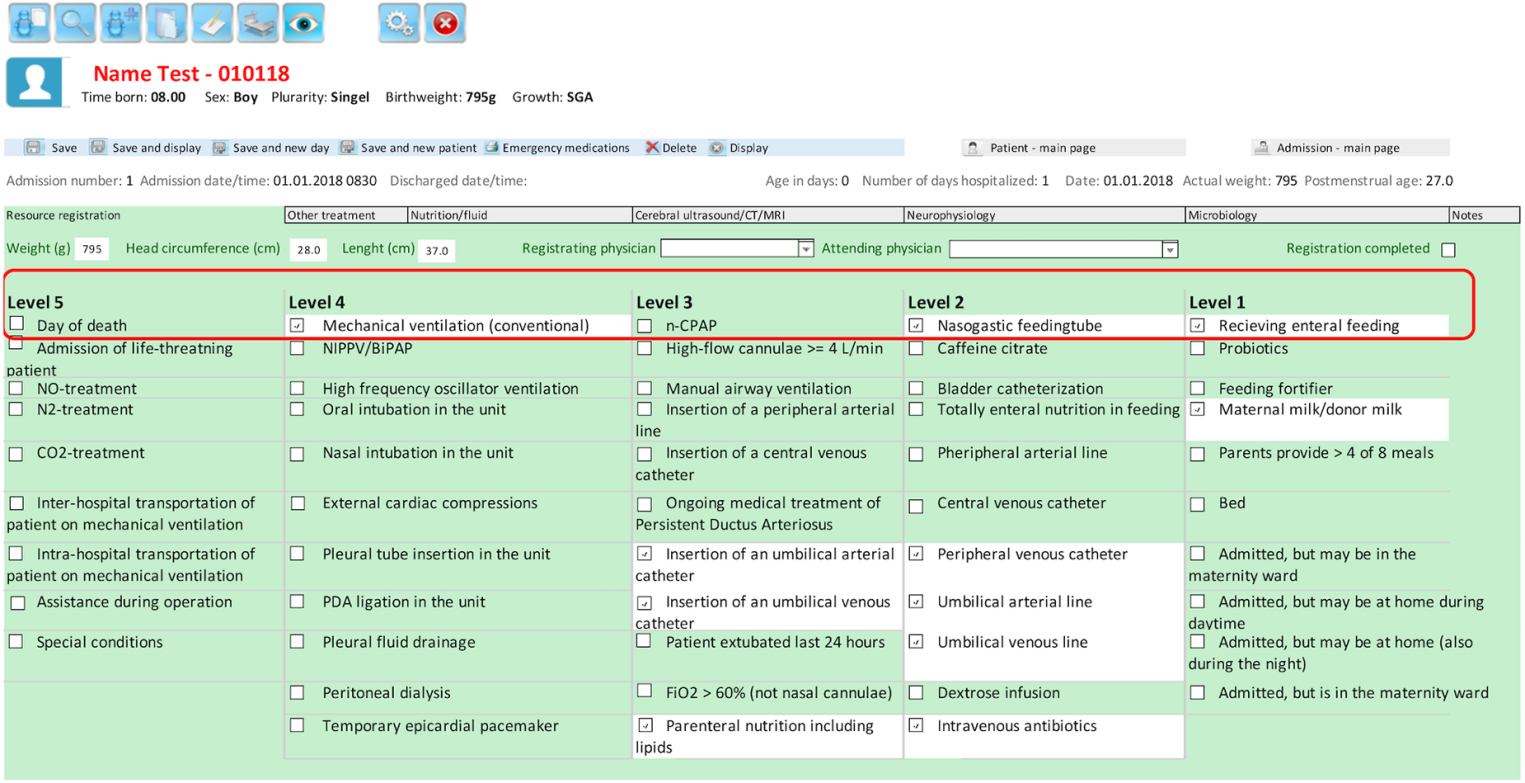
Screenshot of part of the daily resource registration form in the Norwegian neonatal network database (the patient classification system, with levels 1–5). This form was translated into English from Norwegian.

The patient volume of a given NICU on a given day was defined as “the number of all infants staying in the unit,” not only those born below 26 weeks GA and included in our study. Unit acuity was defined as the “intensity of nursing care needed by the patients” in a given NICU on a given day and was calculated based on daily resource registration in the NNN (shown in Figure 1). Each day, patient care and individual treatment procedures are recorded in the NNN database. Patients are classified into five levels, similar to the Vermont Oxford Network researcher’s classification (20). Levels 1 and 2 represent patients with low acuity who require basic monitoring and care. Level 3 represents patients receiving breathing assistance with nasal continuous airway pressure and often drug therapy. These infants require frequent monitoring. Level 4 typically represents patients on MV requiring continuous monitoring, and Level 5 represents patients requiring the highest level of intensive care treatment and surveillance. The coding accuracy for the patient classification variable is considered high, as each hospitalized newborn is registered in the NNN each day, and there is little room for individual interpretation of each newborn’s clinical condition. The total acuity in one NICU for each day was calculated based on an estimation of the need for nursing, as described elsewhere (7). Patient volume and unit acuity were calculated for each day in the study period and defined as a low, normal, or high unit workload based on standard deviations. We also extracted the number of patients admitted and discharged each day, as these patients often require more resources. Furthermore, unit workloads were explored at three time points that are considered important in an EP infant’s course of treatment:

(i) the infant’s day of birth,
(ii) the day of the first extubation attempt, and
(iii) the week after the first extubation attempt.

Moreover, if the infant was reintubated, the workload on the day of reintubation was explored. We chose to explore the unit workload on the infant’s day of birth (DOB), as the DOB is considered important related to prior research about the importance of the golden hour, suggesting that interventions performed in the first minutes after birth may have long-term consequences in addition to short-term effects on the rate and quality of survival of EP infants (21, 22).

### 2.4. Exposure: weekdays and summer holidays

To distinguish weekdays from weekends, weekends were defined as Saturdays and Sundays, as this is the most common weekend definition in “off-shift” research (23). Summer days were defined from July to August, as this is the most common period for annual leave among Norwegian healthcare professionals.

### 2.5. Outcomes associated with exposure (workload, weekend, summer holiday)

The primary outcomes were the duration of MV until the first planned extubation attempt and the outcome of this attempt. Extubation success was defined as no reintubation within 72 h.

Secondary outcomes were causes of reintubation and short-term respiratory morbidity of the infants reintubated within 72 h. The causes of reintubation and variables relevant to the ventilation treatment provided six hours before and after the reintubation event were extracted from the medical records to enable a description of short-term respiratory morbidity. The pre-reintubation variables extracted included the mode of non-invasive ventilation, such as positive end-expiratory pressure, and fraction of inspired oxygen (FiO_2_) administered. The post-reintubation variables included ventilator modes and settings, such as peak inspiratory pressure, mean positive airway pressure, and FiO_2_. In addition, blood gas variables before and after reintubation were extracted.

### 2.6. Statistical analysis

Data were expressed as means with 95% confidence intervals or standard deviations (SDs), medians with 25th and 75th percentiles (the interquartile range), or numbers with proportions (%). For patient volume and unit acuity, *z*-scores for each NICU were calculated for the total of the six years studied because stratified analyses did not show a distinctive change in patient volume or unit acuity over these years. *Z*-scores were calculated as the association between each day’s patient volume and unit acuity as measured by standard deviations from the mean. According to this, each day was defined as normal if the *z*-score was ±1 SD, high if the *z*-score was >+1 SD, and low if the *z*-score was <−1 SD. Depending on the variable distribution, we examined unadjusted associations between the outcome and exposure variables using Kruskal–Wallis and logistic regression analyses with unit workloads as independent variables. In the regression analysis, days with low patient volume or low unit acuity were used as the reference groups. All statistical analyses were performed using Stata/MP (2019, Stata Statistical Software: Release 16. College Station, TX: StataCorp LLC). The threshold for statistical significance was set at *p* < 0.05.

## 3. Results

**Figure 2:**
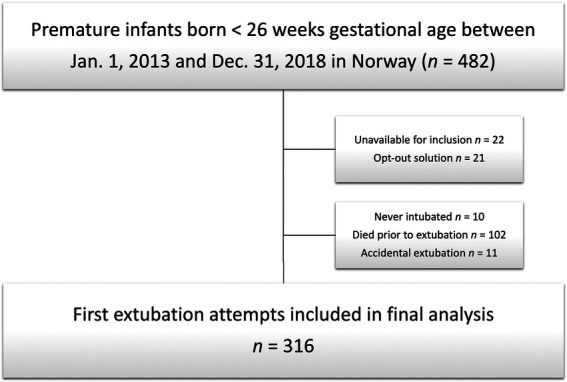
Flowchart of included infants.

Of 482 infants with GA <26 weeks admitted to a NICU during the study period, 43 (9%) infants were excluded, as the mother’s address could not be verified or the mother chose to opt out. Additionally, 10 (2%) infants were excluded because they had never been intubated during admission. Furthermore, 102 (21%) infants who died prior to the first extubation attempt, and 11 (2%) who had an identified accidental extubation were excluded. In the final analysis, 316 first extubation attempts were included (Figure 2). The first extubation attempts were performed in 11 different Norwegian NICUs. Table 1 presents the characteristics of the study population.

**Table 1:** Characteristics of the study population.

Variable	Value
No. of infants	316
GA, weeks, mean (SD)	24.5 (0.8)
Birth weight, mean (SD)	667.8 (136)
Male, *n* (%)	160 (51)
Small for GA, *n* (%)	60 (19)
Caesarean delivery, *n* (%)	103 (33)
Apgar <5 at 5 min of age, *n* (%)	60 (19)
CRIB II score >14, *n* (%)^1^	172/304 (57)
ANS any exposure, *n* (%)^2^	298 (94)
ANS complete course, *n* (%)	183/300 (61)
Surfactant administered prior to 30 min of age, *n* (%)^3^	309/314 (98)
Transport prior to extubation attempt, *n* (%)	20 (6)

GA, gestational age; SD, standard deviation; CRIB, Clinical Risk Index for Babies; ANS, antenatal steroid; NICU, neonatal intensive care unit.

^1^ CRIB II scores were not registered in 12 infants (3.8%).

^2^ ANS complete course: defined as when the first dose was administered at least 24 h before birth. The time of the first dose was not registered in 16 (5%) infants.

^3^ The time of surfactant administration was not registered in two (0.6%) infants.

The associations between the exposure variables and primary outcomes are presented in Table 2. Most of the infants had their first extubation attempt on a day categorized by normal patient volume and normal unit acuity. There was no statistical difference in the outcomes if the infant was extubated on a day with low, normal, or high patient volume or unit acuity. Additionally, there was no association between patient volume and unit acuity in the week after the first extubation attempt with the duration of MV or extubation success (data not shown).

**Table 2:** The association of patient volume, unit acuity, and weekdays with duration of mechanical ventilation and extubation success, *n* = 316.

Variable	Median days with MV until first extubation attempt (IQR)	*P*	Extubation success, *n*/*N* (%)	*P*
Patient volume on day of birth*				
Low^1^	5 (2–17)		17/27 (63)	
Normal	6 (3–19)		113/203 (56)	0.48
High	6 (2–16)	0.75	43/86 (50)	0.24
Patient volume on day of first extubation attempt*				
Low^1^	6 (3–22)		22/37 (59)	
Normal	6 (2–17)		116/214 (54)	0.56
High	6 (2–17)	0.95	35/65 (54)	0.59
Unit acuity on day of birth*				
Low^1^	6 (2–21)		14/20 (70)	
Normal	6 (3–19)		108/193 (56)	0.23
High	6 (3–16)	0.92	51/103 (50)	0.09
Unit acuity on day of first extubation attempt*				
Low^1^	7 (3–23)		19/32 (59)	
Normal	6 (3–17)		107/198 (54)	0.58
High	6 (2–17)	0.88	47/86 (55)	0.65
**Weekday/month of first extubation attempt**				
Monday–Friday	6 (2–18)		129/247 (52)	
Saturday–Sunday	5 (3–17)	0.78	44/69 (64)	0.14
September–June	6 (2–17)		148/265 (56)	
July–August	5 (3–21)	0.67	25/51 (49)	0.44

MV, Mechanical ventilation; IQR, interquartile range.

* Based on *z*-scores for each unit in the study period (January 1, 2013–December 31, 2018). Normal if the *z*-score was +−1 SD, high if the *z*-score was >+1 SD, and low if the *z*-score was <−1 SD.

^1^ Used as a reference group in the regression analysis.

We found that 86 (27%) infants were extubated on days classified as high workload (acuity), while 32 (10%) were extubated on days classified as low workload (acuity) (data not shown).

There were 247 (78%) infants who had their first extubation attempt on a weekday, while 69 (22%) experienced their first attempt on a weekend. Extubation was more often attempted on weekdays compared to Sundays (with a factor of 1.4–1.9, *p *≤ 0.01–0.03). Moreover, 51 (16%) infants had their first extubation attempt on a day categorized as a summer holiday. There was no statistical difference in the duration of MV or extubation success between weekdays and weekends, and no statistical difference in these outcomes if it was a summer holiday compared with other days during the year.

A total of 143 (45%) infants experienced reintubation within 72 h after the first extubation attempt, as described elsewhere (24). The documented causes of reintubation are presented in Table 3. The analyses showed no statistical associations between causes of reintubation and weekends, summer holidays, or unit workloads. For the reintubated infants, the short-term respiratory morbidity and associations with the unit workloads are presented in Table 4. There were borderline significant higher pre-reintubation FiO_2_ between days with a high patient volume and days with a low patient volume, and higher post-reintubation pCO_2_ on days with a normal patient volume compared with those with a low patient volume. No other differences in the pre- and post-reintubation variables were found. Analyses of differences in unit acuity on the day of reintubation revealed similar results.

**Table 3:** Causes of reintubation and associations with patient volume, unit acuity, and the weekday the infant was reintubated.

Variable	Causes of reintubations (within 72 h), *n* = 142^1^									
	Apnea		WOB		High pCO_2_		High FiO_2_		Sepsis	
	*n*	% (95% CI)	*n*	% (95% CI)	*n*	% (95% CI)	*n*	% (95% CI)	*n*	% (95% CI)
Causes of reintubation (all)	68	48	31	22	12	8	23	16	8	6
Patient volume on the day of reintubation*										
Low	6	35 (17–60)	7	41 (21–65)	1	6 (1–32)	1	6 (1–32)	2	11 (3–37)
Normal	55	53 (43–62)	17	16 (10–25)	10	10 (5–17)	16	15 (9–24)	6	6 (3–12)
High	7	33 (17–55)	7	33 (17–56)	1	5 (1–27)	6	28 (13–50)	0	0 (0)
Unit acuity on the day of reintubation*										
Low	6	46 (22–72)	5	38 (17–66)	1	8 (1–39)	1	8 (1–39)	0	0 (0)
Normal	49	50 (40–60)	17	17 (11–26)	9	9 (5–17)	16	16 (10–25)	7	7 (3–14)
High	13	42 (26–60)	9	29 (16–47)	2	6 (2–23)	6	19 (9–37)	1	3 (0–20)
**Weekday/month when reintubated**										
Monday–Friday	45	46 (37–56)	20	20 (14–30)	9	9 (5–17)	17	17 (11–26)	6	6 (3–13)
Saturday–Sunday	23	51 (37–65)	11	24 (14–39)	3	7 (2–19)	6	13 (3–13)	2	4 (1–16)
September–June	52	45 (36–54)	26	22 (16–31)	11	9 (5–16)	21	18 (12–26)	6	5 (2–11)
July–August	16	62 (42–78)	5	19 (8–39)	1	4 (1–23)	2	8 (2–26)	2	8 (2–26)

WOB, Work of breathing; O2, need for a high percentage of oxygen %; CI, confidence interval.

* Based on *z*-scores for each unit in the study period (1.1.2013–31.12.2018). Normal if the *z*-score was +−1 SD, high if the *z*-score was >+1 SD, and low if the *z*-score was <−1 SD.

^1^ Missing the cause of reintubation for one (0.7%) infant.

**Table 4:** Indicators of short-term respiratory morbidity for infants reintubated and associations with patient volume, *n* = 143.

Variable	Patient volume on the day of reintubation*			*P*-value	
	Low	Normal	High	Normal vs. low patient volume	High vs. low patient volume
**Pre-reintubation variable**					
PEEP^1^, mean (SD)	7.1 (0.8)	7.0 (1.0)	6.5 (0.6)	0.49	0.07
Oxygen^1^^,^^2^, median (IQR)	44 (32–48)	42 (32–52)	52 (42–68)	0.90	0.05
pH, median (IQR)	7.23 (7.20–7.30)	7.19 (7.14–7.25)	7.23 (7.15–7.27)	0.14	0.62
pCO_2_^4^, median (IQR)	7.4 (7.0–8.9)	8.4 (7.3–9.9)	8.8 (7.9–10.2)	0.24	0.24
BE, median (IQR)	−3.4 (−7.1 to 0.7)	−4.4 (−6.9 to −0.8)	−1.6 (−6 to 2)	0.69	0.42
**Post-reintubation variable**					
MAP^3^, median (IQR)	9 (9–10)	9 (8–10)	10 (9–12)	0.80	0.19
Oxygen^2^^,^^3^, median (IQR)	34 (28–38)	30 (24–37)	33 (27–42)	0.66	0.43
pH, median (IQR)	7.30 (7.18–7.37)	7.25 (7.18–7.31)	7.30 (7.19–7.35)	0.19	0.55
pCO_2_^4^, mean (SD)	6.7 (1.4)	7.6 (1.7)	7.3 (1.8)	0.05	0.29
BE, mean (SD)	−3.8 (4.9)	−3.4 (5.6)	−2.8 (7.0)	0.84	0.64
RSS^3^, median (IQR)	3.0 (2.5–3.7)	2.8 (2.1–3.6)	2.9 (2.5–5.0)	0.71	0.25
MV days after reintubation, median (IQR)	9 (4–15)	9 (5–16)	8 (5–14)	0.54	0.78

PEEP, positive end expiratory pressure; SD, standard deviation; IQR, interquartile range; BE, base excess; MAP, mean airway pressure; RSS, respiratory severity score; MV, mechanical ventilation.

* Based on *z*-scores for each unit in the study period (January 1, 2013–December 31, 2018). Normal if the *z*-score was +−1 SD, high if the *z*-score was >+1 SD, and low if the *z*-score was <−1 SD.

^1^ Mean values for the last 6 h before reintubation.

^2^ Administered oxygen as a percentage.

^3^ Mean values in the first 6 h after reintubation.

^4^ Values in kPa, kilopascals.

The results from all pre- and post-reintubation variables explored are provided in Supplementary Tables S1 (patient volume) and S2 (unit acuity).

## 4. Discussion

In this national cohort of EP infants <26 weeks GA, unit workloads, weekdays, or summer holiday did not affect the duration of MV until the first extubation attempt or the outcome of the extubation attempt. In addition, for infants reintubated within 72 h, the organizational factors explored did not affect the causes of reintubation or the indicators of respiratory morbidity before or after reintubation. To our knowledge, this is the first study exploring NICU characteristics and outcomes related to the first extubation attempt among EP infants <26 weeks GA.

The results indicating that workload does not affect extubation outcome and reasons for reintubation due not take into account that some NICUs might have a high workload as their “normal” compared to others. High workload (+1 SD) describe approximately the 85th percentile of workloads in each NICU. It is also possible that there might be a non-linear effect on care from workloads. Both low and high workloads might have a positive or negative effect on care. Extreme high workloads could invoke resilience as emergency procedures supplying more personnel.

Our data suggest that clinicians might be more inclined to extubate on days with high workload since 27% were extubated in the 15% of the days with the highest workload. Interestingly, the outcome of the extubation attempt was not influenced by workload.

Our results may have two potential interpretations. First, the results could suggest that the level of standard staffing (or short-term increased staffing) in Norwegian NICUs is sufficient, regardless of fluctuations in patient volume, unit acuity, weekends, and summer holiday. Alternatively, respiratory care of the most vulnerable infants <26 weeks GA might be the least affected and most protected when workload is high. One might speculate that tight and continuous observation and monitoring of EP infants <26 weeks GA on MV is resilient to fluctuations in workloads in the NICU. Resilience in healthcare has been defined as “the capacity to adapt to challenges and changes at different system levels, to maintain high quality care” (25). Resilience at the clinical level (micro level) could have potential negative consequences in other aims of care in the NICU. Resilience at the level of the NICU (meso level) might include increasing staff on short notice. Possible negative consequences of this could be increased personnel burnout and turnover. Resilience at hospital level (macro level) would be sufficient standard staffing for high workload days, with its financial consequences. Our data does not differentiate level of resilience and potential unwanted consequences.

Our database did not include the actual number of healthcare professionals on call in each participating unit on each day. It also did not include the hours actually worked by physicians and nurses. For instance, individual healthcare personnel might make an extra effort to compensate for the higher unit workload and work overtime if needed. The nursing overtime ratio and unit occupancy have been associated with medical incidents, nosocomial infections, and unplanned extubation events (26–28).

The results of our study indicate that healthcare personnel in Norwegian NICUs were able to deliver high-quality short-term respiratory care independent of unit workloads. However, we speculate whether resources used to maintain high quality in fundamental short-term outcomes aimed at airways and breathing could have come at the expense of attention to other important assignments. Tubbs-Cooley et al. determined that high workloads of NICU nurses were significantly associated with missed nursing care, e.g., missed hourly intravenous site assessments, oral feedings, and parental involvement (29). Hence, the long-term consequences of higher workloads and missed care for infants, parents, and healthcare professionals are uncertain.

Our finding that there was no weekend or summer holiday association is comparable to the results of a large cohort study from the National Institute of Child Health and Human Development Neonatal Research Network database. They found little effect on the risks of death and morbidity among very low birth weight infants born on weekends or during the months of July and August (30). However, our study identified fewer extubation attempts on weekends compared to weekdays. This finding may indicate that extubation attempts were postponed from the weekends and that available staffing on weekends might influence judgments related to the timing of extubation. A lower tendency to extubate on weekends could be a contributing factor to prolonged duration of MV. Over the last decades, the weekend effect has been analyzed and discussed in several studies in both adult and maternal-neonatal settings (31–35). Still, the weekend phenomenon is not yet fully understood, emphasizing the need for further studies exploring actual weekend staffing in relation to respiratory neonatal outcomes.

Furthermore, in this study we have explored several indicators of short-term respiratory morbidity for the infants who were reintubated within 72 h after the extubation attempt. Our results did not indicate that infants reintubated on days with high workload was sicker or needed higher level of respiratory support compared with infants reintubated on days with normal or low workloads. However, we found a borderline significant higher pre-reintubation FiO_2_ that could be implied as an indicator of infant stress related to a busy unit. Nevertheless, these results must be interpreted with caution with respect to possible confounders, whereas both patient factors and differences in extubation practice among units could influence these results. The choice and management of the post-extubation therapy might be of importance when it comes to extubation success. In a previous publication exploring predictors of successful extubation carried out on the exact same population we showed that all of these infants were predominantly treated with nasal continuous positive airway pressure immediately after extubation (24).

Our study has certain limitations. First, we included patients treated in 11 different NICUs in Norway and were unable to collect the existing staffing levels, seniority, and experience levels of staff present in the unit on each day. In addition, Norwegian NICUs are small resulting in lack of statistical power to control for cluster effects. Second, we were unable to describe fluctuations in workloads during the day. Previous studies have identified higher odds of mortality for infants admitted to the NICU at night compared to the daytime (36). However, a recent study examining overnight extubation was not able to identify differences in success rates between day and night shifts (37). Future studies investigating unit workloads in relation to actual staffing levels and healthcare experiences are needed to further explore the complex contexts of the first extubation events among EP infants <26 weeks GA. Still, there is a lack of a standard method for modeling unit workloads, and the description of a workload effect depends on several factors, including its measure and definition. We considered two measures of unit workload: patient volume and unit acuity. Our calculations were based on *z*-scores for the total of six years examined. Furthermore, unit acuity may be a more meaningful measure of workloads, as a higher patient volume with relatively few infants at the highest patient levels places different requests on a unit compared with lower patient volumes with a relatively high number of infants demanding intensive care treatment.

The strength of this study is the prospective collection of data on a daily basis by the NNN and the inclusion of a large population-based sample of EP infants <26 weeks GA. The completeness of the variables allowed us to explore unit workloads on days perceived as critical in these vulnerable infants’ courses of treatment in the NICU. Several neonatal networks (e.g., the Vermont Oxford Network, Neonatal Research Network, Italian Neonatal Network, and others) collect data on an individual infant level, and research into the treatment and outcomes of premature infants has expanded, partially due to these large multicenter databases (38). However, few studies using data from neonatal databases address features of the environment where neonatal care takes place, e.g., the unit workload.

In conclusion, we found that there was no association between unit workloads and weekday/summer holiday with the duration of MV until the first extubation attempt and the result of the attempt. The data, containing daily registered measures of acuity for each infant, made it possible to calculate objective indicators of NICU workloads in addition to patient volume. Our results may suggest that the potential threat to short-term respiratory morbidity associated with total patient burden is alleviated by resilience. Further research is needed to examine potential negative consequences for infants and staff in the NICU.

## Data Availability

The datasets presented in this article are not readily available because they contain information that could compromise the privacy of research participants. The data that support the findings of this study are available from the corresponding author, MOO, upon reasonable request. Requests to access the datasets should be directed to *mari.oma.ohnstad@ldh.no*
