# Supplementary material for: Associations between unit workloads and outcomes of first extubation attempts in extremely premature infants below a gestational age of 26 weeks
Source: Front Pediatr. 2023 Mar 17;11:1090701. doi: 10.3389/fped.2023.1090701 (PMC10064049; doi:10.3389/fped.2023.1090701)
Supplement: Supplementary file 1 [file Table1.docx]

| **Supplementary Table 1.** Respiratory treatment before and after reintubation and association with patient volume on the day of reintubation, *n* = 143 | | | | | |
| --- | --- | --- | --- | --- | --- |
| Variable | Patient volume* | | | *P-*value | |
|  | Low | Normal | High | Normal vs. low | High vs. low |
| Pre-reintubation variable |  |  |  |  |  |
| BiPAP, *n* (%) | 8 (47) | 51(50) | 14 (67) | 0.85 | 0.23 |
| NCPAP, *n* (%) | 9 (53) | 52 (50) | 7 (33) |  |  |
| Last registered PEEP,  mean (SD) | 7.1 (1.1) | 7.0 (0.9) | 6.6 (0.7) | 0.75 | 0.12 |
| Mean PEEP last 6 h,  mean (SD) | 7.1 (0.8) | 7.0 (1.0) | 6.5 (0.6) | 0.49 | 0.07 |
| Last registered FiO_2_,  median (IQR) | 50 (33–60) | 49 (37–70) | 59 (40–81) | 0.43 | 0.17 |
| Mean FiO_2_ last 6 h,  median (IQR) | 44 (32–48) | 42 (32–52) | 52 (42–68) | 0.90 | 0.05 |
| pH, median (IQR)^1^ | 7.23 (7.20–7.30) | 7.19 (7.14–7.25) | 7.23 (7.15–7.27) | 0.14 | 0.62 |
| pCO2, median (IQR)^1,2^ | 7.4 (7.0–8.9) | 8.4 (7.3–9.9) | 8.8 (7.9–10.2) | 0.24 | 0.24 |
| BE, median (IQR)^1^ | -3.4 (-7.1–0.7) | -4.4 (-6.9– -0.8) | -1.6 (-6–2) | 0.69 | 0.42 |
| Post-reintubation variable |  |  |  |  |  |
| CV, *n* (%) | 16 (94) | 93 (89) | 13 (62) | 0.60 | 0.004 |
| HFOV, *n* (%) | 1 (6) | 11 (11) | 8 (38) |  |  |
| First registered PIP,  mean (SD) | 19 (2.3) | 19 (4.1) | 19 (3.6) | 0.94 | 0.88 |
| Mean PIP first 6 h,  mean (SD) | 18 (2.1) | 19 (3.3) | 18 (2.8) | 0.79 | 0.71 |
| First registered PEEP,  mean (SD) | 5.8 (0.8) | 5.7 (0.7) | 5.8 (0.8) | 0.98 | 0.73 |
| Mean PEEP first 6 h,  mean (SD) | 5.8 (0.8) | 5.7 (0.7) | 5.8 (0.7) | 0.91 | 0.71 |
| First registered MAP,  median (IQR) | 9 (9–10) | 9 (8–10) | 10 (9–12) | 0.48 | 0.23 |
| Mean MAP first 6 h,  median (IQR) | 9 (9–10) | 9 (8–10) | 10 (9–12) | 0.80 | 0.19 |
| First registered FiO_2_,  median (IQR) | 35 (29–50) | 32 (24–45) | 36 (26–55) | 0.63 | 0.56 |
| Mean FiO_2_ first 6 h,  median (IQR) | 34 (28–38) | 30 (24–37) | 33 (27–42) | 0.66 | 0.43 |
| pH, median (IQR)^1^ | 7.30 (7.18–7.37) | 7.25 (7.18–7.31) | 7.30 (7.19–7.35) | 0.19 | 0.55 |
| pCO2, mean (SD)^1,2^ | 6.7 (1.4) | 7.6 (1.7) | 7.3 (1.8) | 0.05 | 0.29 |
| BE, mean (SD)^1^ | -3.8 (4.9) | -3.4 (5.6) | -2.8 (7.0) | 0.84 | 0.64 |
| RSS, median (IQR)^3^ | 3.0 (2.5–3.7) | 2.8 (2.1–3.6) | 2.9 (2.5–5.0) | 0.71 | 0.25 |
| MV course, median  (IQR), days | 9 (4–15) | 9 (5–16) | 8 (5–14) | 0.54 | 0.78 |
| *BIPAP*, bi-level positive airway pressure; *NCPAP*, nasal continuous positive airway pressure; *SD*, standard deviation; *PEEP*, positive end expiratory pressure; *FiO_2_*, fraction of inspired oxygen; *IQR*, interquartile range; *BE*, base excess; *CV*, conventional ventilation; *HFOV*, high-frequency oscillator ventilation; *PIP*, positive inspiratory pressure; *MAP*, mean airway pressure; *RSS*, respiratory severity score; *MV*, mechanical ventilation  *Based on z-scores for each unit in the study period (1.1.2013–31.12-2018). Normal if the z-score was +-1 SD, high if the z-score was > +1 SD, and low if the z-score was < -1 SD.  ^1^Measured in arterial, capillary, or venous blood samples.  ^2^pCO2 values in kilopascals (*7.50062 provides values in millimeters of mercury).  ^3^RSS was calculated as a product of MAP and a fraction of inspired oxygen. RSS was calculated based on the last 6 h after reintubation. | | | | | |
