# Supplementary material for: Associations between unit workloads and outcomes of first extubation attempts in extremely premature infants below a gestational age of 26 weeks
Source: Front Pediatr. 2023 Mar 17;11:1090701. doi: 10.3389/fped.2023.1090701 (PMC10064049; doi:10.3389/fped.2023.1090701)
Supplement: Supplementary file 2 [file Table2.docx]

| **Supplementary Table 2.** Respiratory treatment before and after reintubation and association with unit acuity on the day of reintubation, *n* = 143 | | | | | |
| --- | --- | --- | --- | --- | --- |
| Variable | Unit acuity^*^ | | | *P*-value | |
|  | Low | Normal | High | Normal vs. low | High vs. low |
| Pre-reintubation variable |  |  |  |  |  |
| BiPAP, *n* (%) | 8 (62) | 49 (50) | 16 (53) | 0.44 | 0.63 |
| NCPAP, *n* (%) | 5 (38) | 49 (50) | 14 (47) |  |  |
| Last registered PEEP,  mean (SD) | 7.2 (0.8) | 6.9 (0.9) | 6.8 (1.0) | 0.29 | 0.13 |
| Mean PEEP last 6 h,  mean (SD) | 7.2 (0.8) | 6.9 (1.0) | 6.8 (0.8) | 0.41 | 0.25 |
| Last registered FiO_2_,  median (IQR) | 40 (38–50) | 50 (37–70) | 55 (33–85) | 0.28 | 0.15 |
| Mean FiO_2_ last 6 h,  median (IQR) | 42 (36–46) | 43 (33–51) | 52 (34–62) | 0.77 | 0.18 |
| pH, median (IQR) | 7.20 (7.10–7.31) | 7.21 (7.14–7.25) | 7.21 (7.15–7.26) | 1.0 | 0.64 |
| pCO2, median (IQR) | 8.9 (7.0–10.1) | 8.4 (7.4–9.9) | 8.6 (6.6–9.4) | 0.69 | 0.93 |
| BE, median (IQR) | -6.0 (-8.0–0.6) | -4.0 (-6.3–-0.3) | -4.4 (-7.0–0.0) | 0.94 | 0.94 |
| Post-reintubation variable |  |  |  |  |  |
| CV, *n* (%) | 13 (100) | 88 (90) | 21 (68) | 0.31 | 0.004 |
| HFOV, *n* (%) | 0 (0) | 10 (10) | 10 (32) |  |  |
| First registered PIP,  mean (SD) | 18.8 (1.8) | 18.6 (4.1) | 18.3 (3.8) | 0.85 | 0.67 |
| Mean PIP first 6 h,  mean (SD) | 18.3 (1.6) | 18.7 (3.1) | 18.2 (3.6) | 0.72 | 0.88 |
| First registered PEEP,  mean (SD) | 5.5 (0.5) | 5.8 (0.8) | 5.8 (0.8) | 0.14 | 0.21 |
| Mean PEEP first 6 h,  mean (SD) | 5.5 (0.5) | 5.7 (0.7) | 5.9 (0.8) | 0.24 | 0.12 |
| First registered MAP,  median (IQR) | 9 (8–10) | 9 (8–10) | 10 (9–12) | 0.35 | 0.03 |
| Mean MAP first 6 h,  median (IQR) | 9 (8–9) | 9 (9–10) | 10 (9–12) | 0.17 | 0.02 |
| First registered FiO_2_,  median (IQR) | 30 (21–40) | 35 (25–47) | 32 (25–48) | 0.13 | 0.29 |
| Mean FiO_2_ first 6 h,  median (IQR) | 28 (23–34) | 31 (24–38) | 33 (25–40) | 0.28 | 0.25 |
| pH, median (IQR) | 7.26 (7.21–7.31) | 7.25 (7.18–7.31) | 7.29 (7.20–7.32) | 0.49 | 0.68 |
| pCO2, mean (SD) | 6.8 (1.5) | 7.6 (1.7) | 7.2 (2.0) | 0.13 | 0.46 |
| BE, mean (SD) | -4.3 (4.0) | -3.1 (5.9) | -3.8 (5.7) | 0.49 | 0.79 |
| RSS, median (IQR) | 2.5 (1.9–3.0) | 2.8 (2.2–3.8) | 2.9 (2.5–4.4) | 0.19 | 0.10 |
| MV course, median  (IQR), days | 8 (3–11) | 9 (5–16) | 11 (5–16) | 0.47 | 0.57 |
| *BIPAP*, bi-level positive airway pressure; *NCPAP*, nasal continuous positive airway pressure; *SD*, standard deviation; *PEEP*, positive end expiratory pressure; *FiO_2_*, fraction of inspired oxygen; *IQR*, interquartile range; *BE*, base excess; *CV*, conventional ventilation; *HFOV*, high-frequency oscillator ventilation; *PIP*, positive inspiratory pressure; *MAP*, mean airway pressure; *RSS*, respiratory severity score; *MV*, mechanical ventilation  *Based on z-scores for each unit in the study period (1.1.2013–31.12.2018). Normal if the z-score was +-1 SD, high if the z-score was > +1 SD, and low if the z-score was < -1 SD.  ^1^Measured in arterial, capillary, or venous blood samples.  ^2^pCO2-values in kilopascals (*7.50062 provide values in millimeters of mercury).  ^3^RSS was calculated as a product of MAP and a fraction of inspired oxygen. RSS was calculated based on the last 6 h after reintubation. | | | | | |
